# Supplementary material for: Evidence supported by Mendelian randomization: impact on inflammatory factors in knee osteoarthritis
Source: Front Med (Lausanne). 2024 May 28;11:1382836. doi: 10.3389/fmed.2024.1382836 (PMC11165061; doi:10.3389/fmed.2024.1382836)
Supplement: Supplementary file 1 [file Data_Sheet_1.ZIP › data/GCST90274836_5e6/836.leaveoneout.pdf]

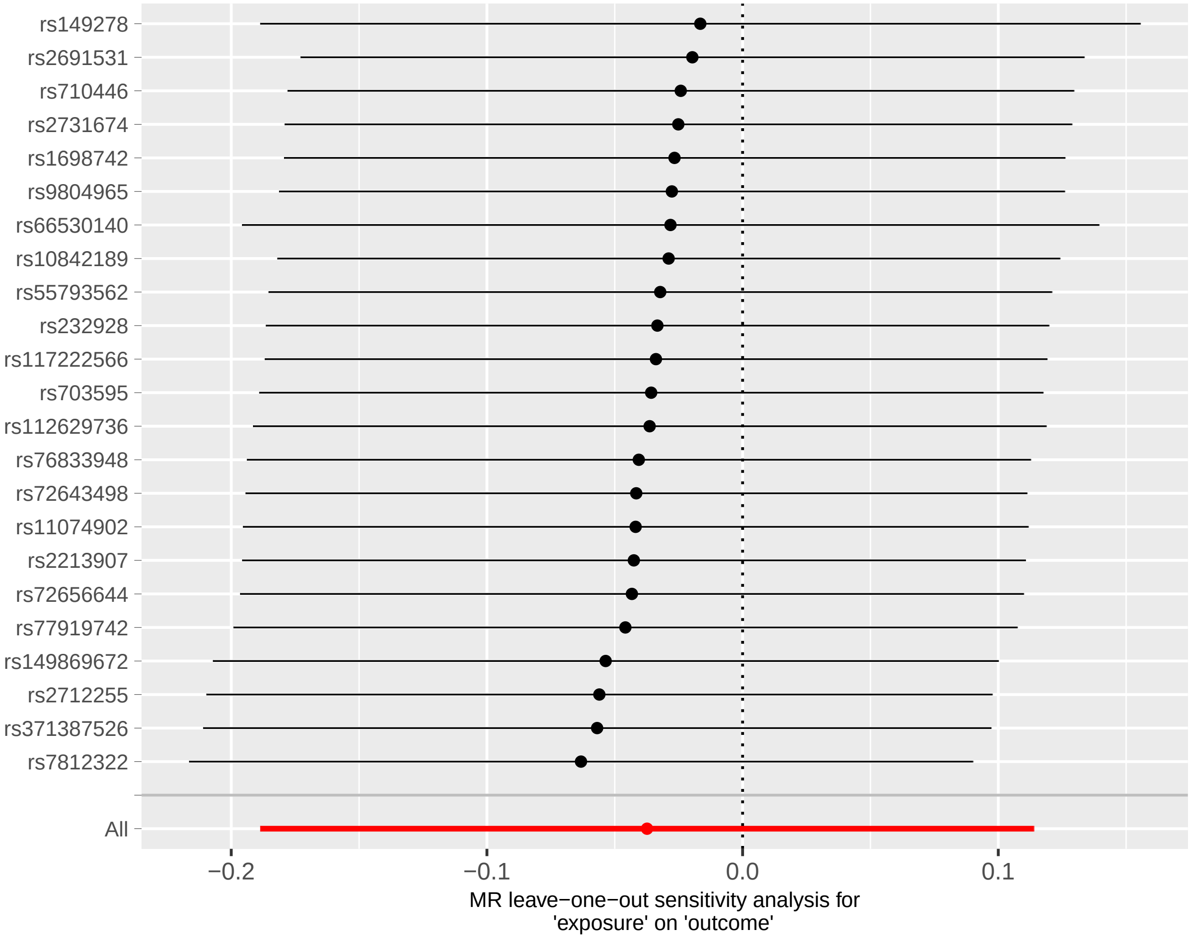
rs149278
rs2691531
rs710446
rs2731674
rs1698742
rs9804965
rs66530140
rs10842189
rs55793562
rs232928
rs117222566
rs703595
rs112629736
rs76833948
rs72643498
rs11074902
rs2213907
rs72656644
rs77919742
rs149869672
rs2712255
rs371387526
rs7812322
All
−0.2
−0.1
0.0
0.1
MR leave−one−out sensitivity analysis for
'exposure' on 'outcome'
